# Supplementary material for: The control of tonic pain by active relief learning
Source: eLife. 2018 Feb 27;7:e31949. doi: 10.7554/eLife.31949 (PMC5843408; doi:10.7554/eLife.31949)
Supplement: Transparent reporting form [file elife-31949-transrepform.pdf]

1st Floor
24 Hills Road
Cambridge CB2 1JP, UK

P 01223 855340
W elifesciences.org
T @elife

eLife's transparent reporting form

We encourage authors to provide detailed information *within their submission* to facilitate the interpretation and replication of experiments. Authors can upload supporting documentation to indicate the use of appropriate reporting guidelines for health-related research (see EQUATOR Network), life science research (see the BioSharing Information Resource), or the ARRIVE guidelines for reporting work involving animal research. Where applicable, authors should refer to any relevant reporting standards documents in this form.

If you have any questions, please consult our Journal Policies and/or contact us: editorial@elifesciences.org.

Sample-size estimation

- You should state whether an appropriate sample size was computed when the study was being designed
- You should state the statistical method of sample size computation and any required assumptions
- If no explicit power analysis was used, you should describe how you decided what sample (replicate) size (number) to use

Please outline where this information can be found within the submission (e.g., sections or figure legends), or explain why this information doesn't apply to your submission:

In Experiment 1, the design was based on an existing study which explored Pavlovian and instrumental learning for simple rewards: O'Doherty et al. Dissociable Roles of Ventral and Dorsal Striatum in Instrumental Conditioning, Science 2004. It used a sample size of n=12, and robustly identified neural correlates of reward prediction errors. Since the ability to identify the behavioural effects of uncertainty is novel, we had no a priori effect sizes to enter into power simulations, so we used a liberal n=19. Experiment 2 aimed to replicate the key findings of Experiment 1, and we used the same analysis methodology. To ensure these results would be robust, we further increased the number of data points within the task, and increased n=23. The fact that the results hold across two experiments (i.e. insofar as Experiment 2 is a 'replication' of Experiment 1), provides a high level of confidence in the findings.

Replicates

- You should report how often each experiment was performed
- You should include a definition of biological versus technical replication
- The data obtained should be provided and sufficient information should be provided to indicate the number of independent biological and/or technical replicates
- If you encountered any outliers, you should describe how these were handled
- Criteria for exclusion/inclusion of data should be clearly stated
- High-throughput sequence data should be uploaded before submission, with a private link for reviewers provided (these are available from both GEO and ArrayExpress)

eLife Sciences Publications, Ltd is a limited liability non-profit non-stock corporation incorporated in the State of Delaware, USA, with company number 5030732, and is registered in the UK with company number FC030576 and branch number BR015634 at the address 1st Floor, 24 Hills Road, Cambridge CB2 1JP | August 2014

Please outline where this information can be found within the submission (e.g., sections or figure legends), or explain why this information doesn't apply to your submission:

Information regarding data collection and stimulation were given throughout the main texts and the Methods section with sufficient details for replication. Experiment 2 in our study is also in essence a replication of Experiment 1, where data was obtained in a different site with a different scanner.

We chose to use open-source software packages for data preprocessing and model fitting, and have included details in the manuscript for replication:
1. Model fitting and comparison: VAB toolbox [https://github.com/MBB-team/VBA-toolbox, forked on github on 31/03/2017],
2. Skin conductance response (SCR) processing: PsPM [http://pspm.sourceforge.net/, v3.1],
3. fMRI data preprocessing: fmriprep [https://github.com/poldracklab/fmriprep, docker build date 09/03/2017],
4. fMRI data analysis: SPM12 [http://www.fil.ion.ucl.ac.uk/spm/software/spm12/, v6842).

We have specified SCR and pain ratings exclusion criteria in the Methods section. For SCR, we followed previous examples to determine whether a response was sufficient by comparing that to a fixed threshold of amplitude 0.02 [Li et al., Nature Neuroscience 2011, Schiller et al., The Journal of Neuroscience 2008]. We excluded an entire session if there were more than 20% trials below the 0.02 amplitude threshold based on previous model fitting experience in our group.

For pain ratings, we excluded one subject because of constant ratings of >90% of all rated trials. This subject's data would produce outliers when fitted to dynamically varying model outputs.

We did not exclude any subject/session from neuroimaging analysis.

There were no high-throughput sequence data in our study.

eLife Sciences Publications, Ltd is a limited liability non-profit non-stock corporation incorporated in the State of Delaware, USA, with company number 5030732, and is registered in the UK with company number FC030576 and branch number BR015634 at the address 1st Floor, 24 Hills Road, Cambridge CB2 1JP | August 2014

Statistical reporting

- Statistical analysis methods should be described and justified
- Raw data should be presented in figures whenever informative to do so (typically when N per group is less than 10)
- For each experiment, you should identify the statistical tests used, exact values of N, definitions of center, methods of multiple test correction, and dispersion and precision measures (e.g., mean, median, SD, SEM, confidence intervals; and, for the major substantive results, a measure of effect size (e.g., Pearson's r, Cohen's d)
- Report exact p-values wherever possible alongside the summary statistics and 95% confidence intervals. These should be reported for all key questions and not only when the p-value is less than 0.05.

Please outline where this information can be found within the submission (e.g., sections or figure legends), or explain why this information doesn't apply to your submission:

Details of statistical analysis can be found in the Methods section. For behavioural data, statistical tests used were indicated in the main text's Results section, sample size N were indicated in both the main text and in figure titles. We used mean, SEM, and Pearson's r throughout the study, which were clearly labeled. p-values of summary statistics were reported rounding to the nearest 3 significant digits, or summarised as <0.001.

For neuroimaging results, we showed our statistical maps at p<0.001 uncorrected, following the convention in the model-based fMRI community. We conducted multiple comparison at whole-brain cluster level FWE-corrected with cutoff at p<0.05, or with small volume correction given strong prior hypothesis in regions of interests. These details were given in the main text and in figure legends. Multiple comparison statistics details were summarised in Table 4 and 5 for the 2 experiments.

(For large datasets, or papers with a very large number of statistical tests, you may upload a single table file with tests, Ns, etc., with reference to sections in the manuscript.)

Group allocation

- Indicate how samples were allocated into experimental groups (in the case of clinical studies, please specify allocation to treatment method); if randomization was used, please also state if restricted randomization was applied
- Indicate if masking was used during group allocation, data collection and/or data analysis

Please outline where this information can be found within the submission (e.g., sections or figure legends), or explain why this information doesn't apply to your submission:

Not applicable, as our study did not have experimental groups.

For within-subject yoking in Experiment 1, we counter-balanced instrumental/Pavlovian sessions to minimise order confounds. This was detailed in the Methods section and Figure 1.

Additional data files ("source data")

eLife Sciences Publications, Ltd is a limited liability non-profit non-stock corporation incorporated in the State of Delaware, USA, with company number 5030732, and is registered in the UK with company number FC030576 and branch number BR015634 at the address 1st Floor, 24 Hills Road, Cambridge CB2 1JP | August 2014

- We encourage you to upload relevant additional data files, such as numerical data that are represented as a graph in a figure, or as a summary table
- Where provided, these should be in the most useful format, and they can be uploaded as "Source data" files linked to a main figure or table
- Include model definition files including the full list of parameters used
- Include code used for data analysis (e.g., R, MatLab)
- Avoid stating that data files are "available upon request"

Please indicate the figures or tables for which source data files have been provided:

Group level statistical maps of our major models can be uploaded to Neurovault. Raw neuroimaging data from all subjects in BIDS format (before preprocessing) can be uploaded to a repository that supports large datasets (Experiment 1: 2.33Gb, Experiment 2: 3.28Gb).

Processed SCRs (in .mat files) and choice data (a single .mat or .csv file) can be uploaded to an online repository.

We have summarised the best-fit parameters of all models used, and detail settings of model fitting in VBA toolbox in Table 2 and 3.

eLife Sciences Publications, Ltd is a limited liability non-profit non-stock corporation incorporated in the State of Delaware, USA, with company number 5030732, and is registered in the UK with company number FC030576 and branch number BR015634 at the address 1st Floor, 24 Hills Road, Cambridge CB2 1JP | August 2014
